# Achievement of High Strength and Ductility in Al–Si–Cu–Mg Alloys by Intermediate Phase Optimization in As-Cast and Heat Treatment Conditions

**DOI:** 10.3390/ma13030647

**Published:** 2020-02-01

**Authors:** Bingrong Zhang, Lingkun Zhang, Zhiming Wang, Anjiang Gao

**Affiliations:** 1 School of Mechanical and Automotive Engineering, Qilu University of Technology (Shandong Academy of Sciences), Jinan 250353, China; brzit@aliyun.com (B.Z.); zhi820426@163.com (Z.W.); 2 Conglin Group Co.Ltd. Longkou, Yantai 265705, China; gaj@conglin.com.cn

**Keywords:** soluble phases, insoluble phases, high strength and ductility, Al–Si–Cu–Mg alloys

## Abstract

In order to obtain high-strength and high-ductility Al–Si–Cu–Mg alloys, the present research is focused on optimizing the composition of soluble phases, the structure and morphology of insoluble phases, and artificial ageing processes. The results show that the best matches, 0.4 wt% Mg and 1.2 wt% Cu in the Al–9Si alloy, avoided the toxic effect of the blocky Al_2_Cu on the mechanical properties of the alloy. The addition of 0.6 wt% Zn modified the morphology of eutectic Si from coarse particles to fine fibrous particles and the texture of Fe-rich phases from acicular β-Fe to blocky π-Fe in the Al–9Si–1.2Cu–0.4Mg-based alloy. With the optimization of the heat treatment parameters, the spherical eutectic Si and the fully fused β-Fe dramatically improved the ultimate tensile strength and elongation to fracture. Compared with the Al–9Si–1.2Cu–0.4Mg-based alloy, the 0.6 wt% Zn modified alloy not only increased the ultimate tensile strength and elongation to fracture of peak ageing but also reduced the time of peak ageing. The following improved combination of higher tensile strength and higher elongation was achieved for 0.6 wt% Zn modified alloy by double-stage ageing: 100 °C × 3 h + 180 °C × 7 h, with mechanical properties of ultimate tensile strength (UTS) of ~371 MPa, yield strength (YS) of ~291 MPa, and elongation to fracture (E%) of ~5.6%.

## 1. Introduction

The contradictory development of high load stress and thin-walled structures of engine cylinder heads limits the application of traditional A356 and A319 materials. In recent years, the research and development of engine materials with high strength and ductility has become a topic of interest [1,2,3]. In Al–Si–Cu–Mg alloys, the existence of intermediate phases can guarantee high strength by blocking dislocation movement, but the ductility is poor. To obtain high strength and ductility of Al–Si–Cu–Mg alloys, the parameters of the intermediate phases (type, size, morphology, and distribution) need to be controlled by the macroprocess. According to their thermal stability, the intermediate phases can be divided into soluble and insoluble phases [4,5].

In Al–Si–Cu–Mg alloys, the soluble phases consist of Mg_2_Si, Al_2_Cu, and Q-Al_5_Cu_2_Mg_8_Si_6_, the mixture ratios of which are dependent on the Cu/Mg ratio [6,7]. The effect of Cu and Mg additions on the soluble phase composition and the improvement of strength in Al–Si alloys have been important topics [8,9,10,11,12]. Sjölander et al. [8] found that the 0.5 wt% Mg addition in the Al–8Si–3Cu alloy promoted the formation of Q-Al_5_Cu_2_Mg_8_Si and dramatically improved the ageing strength compared with the Mg-free alloy counterpart. Taylor et al. [9] found that the (0.3 wt%–0.7 wt%) Mg addition in the Al–8Si alloy promoted the formation of Mg_2_Si, and the increase in peak yield strength was linear up to a Mg concentration of approximately 0.5 wt%, while the increase was greatly reduced when Mg concentrations were continuously added. Wang et al. [10] indicated that precursors of the Al_2_Cu form in Al–Si–Cu alloys at ageing, but the age response per added wt% of Cu is lower in casting alloys. Although the Cu and Mg additions can greatly improve the strength of Al–Si alloys, the increase in strength is associated with the decrease in elongation, as the added reinforcement of intermetallic phases comes at the cost of alloy ductility [13,14].

In the Al–Si–Cu–Mg alloys, the insoluble phases are composed of eutectic Si and Fe-rich phases [15]. Before modification, they appear in the Al matrix in a sharp acicular structure, which is harmful to the mechanical properties, especially to the elongation to fracture [16]. Unlike the dissolution and precipitation strengthening of the soluble phases, insoluble phases can only have a strengthening effect when their morphology and texture are modified. The modification of eutectic Si by Na and Sr has been widely studied [17,18,19,20]. The modified fibrous eutectic Si can effectively avoid the formation of a crack source, but the improved coherence with the Al matrix limits the effective improvement of elongation to fracture. Compared with the modification of eutectic Si, the modification of Fe-rich phases becomes difficult due to their high chemical stability. The chemical composition of Fe-rich phases can be divided into acicular β-Al_5_FeSi and blocky π-Al_5_FeMg_3_Si_6_. The π-Fe with poor thermal stability is decomposed into small β-Fe and Mg atoms in the early stage of solution treatment. Compared with β-Fe, π-Fe has a less harmful effect on the mechanical properties of Al–Si alloys [21,22,23,24].

Ageing processes play an important role in the determination of the intermediate phases in Al–Si–Cu–Mg alloys. Under ageing, the Cu and Mg atoms in the supersaturated Al matrix are enriched in the grain boundary to form the GP zone; GP zone dissolution then occurs, and metastable precipitates begin to nucleate there; the metastable precipitates are continuously merged to form stable precipitates [25]. Although the formation of the GP zone cannot directly improve the strength of the alloy, its homogeneous distribution lays the foundation for mechanical property improvements at peak ageing because it can provide the nucleation sites for the metastable precipitates [26]. To obtain the GP zone with high density and uniform distribution, it is necessary to optimize the initial ageing temperature.

Therefore, this work studies how to achieve better values of both strength and ductility in Al–Si–Cu–Mg alloys throughout the optimization, step by step, of the soluble phase composition, the insoluble phase structure and morphology, and the artificial ageing processes. The mechanical properties of the alloy at each optimization step are reported. The types of soluble phases, the texture and size of insoluble phases, and the fracture surface of the alloy under different artificial ageing processes are analyzed.

## 2. Materials and Methods

### 2.1. Optimization of the Base Alloy with the Best Matches of Cu and Mg

The chemical compositions of the Al–Si–Cu–Mg alloys studied in this work are shown in Table 1 and Table 2, based on an Al–Si9 alloy with different Cu (0 wt%–3 wt%) and Mg (0.2 wt%–0.6 wt%) concentrations. Sr modification and grain refinement were used. Cylindrical rods were cast in a preheated permanent mold. To determine the types of intermediate phases, differential scanning calorimetry (DSC) was performed on a Netzsch DSC404 F3 (NETZSCH Group, Serb, Germany) under a high-purity Ar atmosphere. The specimens were 3 mm in diameter and 10 ± 0.2 mg in weight (the GB/T 19466 Chinese Standard for DSC specimen). High-purity Al_2_O_3_ was used as a reference material, and the baseline was subtracted from the data. In order to clearly detect the endothermic peak of each intermediate phase, the test temperature of 300–700 °C and the test rate of 5 °C/min were selected. Double-stage solution treatment (500 °C × 3 h + 520 °C × 5 h) was conducted in an electrical furnace. After quenching in hot water of 60 °C, all specimens were artificially aged at 180 °C for 9 h. The tensile test specimens with gauge lengths of 144 mm and diameters of 10 mm (the ASTM E-8 standard tensile test specimen size) were machined from the rods after the heat treatment. The tensile tests were carried out on a WDW-300E universal testing machine at a strain rate of 2 mm/min (Maijie Group, Jinan, China). Each test result reported in this work was averaged from three tensile test specimens.

### 2.2. Modification of Insoluble Phases

After optimizing the base alloys with the best matches of Cu and Mg, different contents of Zn (0 wt%–0.9 wt%) were added to modify insoluble phases, i.e., the composition of the eutectic Si and Fe-rich phases, as shown in Table 3. The process of casting and heat treatment were consistent with the processes mentioned above in Section 2.1. The mechanical properties of Zn-modified alloys were tested after heat treatment. Optical microscopy (OM, DM2700M, Leica microsystems Co., Ltd, Wetzlar, Germany) and scanning electron microscopy (SEM, Regulus8220, HITACHI, Tokyo, Japan) with energy dispersive spectroscopy (EDS) were used to analyze the morphology and texture of insoluble phases.

### 2.3. Optimization of Artificial Ageing Processes

After optimizing the Zn-modified alloys with tiny-sized insoluble phases, three types of artificial ageing processes were applied to the optimization of the modified alloy, namely low temperature ageing (90 °C for 0–12 h), high temperature ageing (180 °C for 0–12 h), and double-stage ageing (90 °C for 3 h + 180 °C for 0–9 h). For comparison, three types of artificial ageing processes were also applied to the optimization of the base alloy. Three tensile test bars for each ageing condition were used to ascertain reproducibility. The tensile testing equipment and the strain rate were in agreement with the above. The fracture surfaces of tensile bars of the two alloys under the three artificial ageing processes were analyzed by SEM.

## 3. Results and Discussion

### 3.1. Optimization of the Base Alloy with the Best Matches of Cu and Mg

#### 3.1.1. Effect of Cu and Mg Contents on Soluble Phases in Al–Si–Cu–Mg Alloy

**Figure 1:**
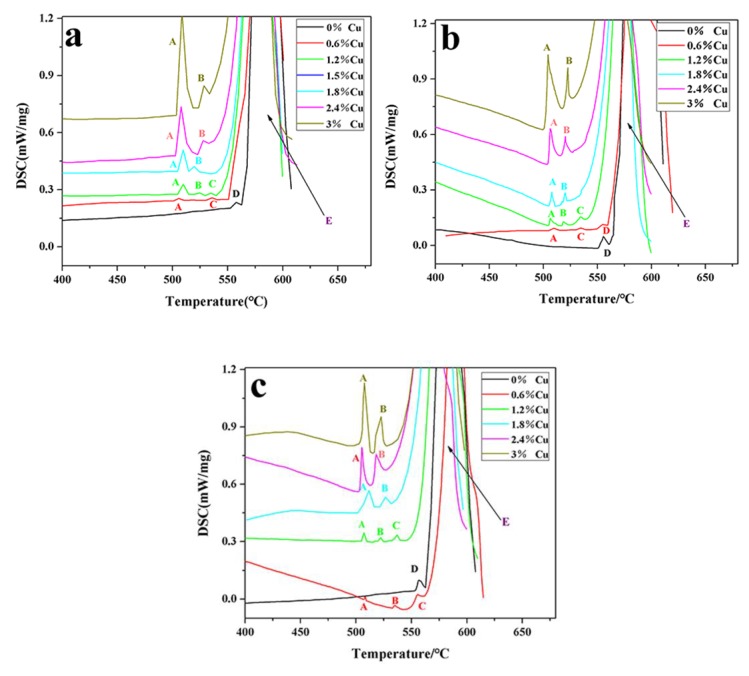
DSC scan of alloys M1 (**a**), M2 (**b**), and M3 (**c**).

DSC scan curves of alloys M1, M2, and M3 are presented in Figure 1a–c. According to the reaction of endothermic peaks (Table 4) deduced by Mondolfo [27], for alloy M1, the soluble phase was only Mg_2_Si (peak D) in Cu-free alloy. When 0.6 wt% Cu was added, the Mg_2_Si phase (peak D) was replaced by eutectic Al_2_Cu (peak A) and the Q phase (peak C). With the increase in Cu content, a small amount of blocky Al_2_Cu (peak B) was formed when 1.2 wt% Cu was added. When the Cu content reached 1.8 wt%, exothermic peak C disappeared, and eutectic Al_2_Cu (peak A) and blocky Al_2_Cu (peak B) became the main soluble phases. After that, the type of soluble phases no longer changed, but the areas of the exothermic peaks corresponding to eutectic Al_2_Cu and blocky Al_2_Cu increased with increasing Cu content. According to the area of each endothermic peak (Table 5), when the content of Cu increased from 1.2 wt% to 3 wt% in alloy M1, the area of endothermic peak A (eutectic Al_2_Cu) and the area of endothermic peak B (blocky Al_2_Cu) were increased from 2.32 J/g and 0.73 J/g to 21.45 J/g and 4.12 J/g, increasing by 19.13 J/g and 3.39 J/g, respectively. This result was in good agreement with Sauvage [10], who pointed out that the increase of Cu content inevitably led to the transformation of soluble phases from Mg-rich phases to Cu-rich phases. When the Cu content reached 2 wt%, the soluble phases consisted of eutectic Al_2_Cu and bulk Al_2_Cu.

**Figure 2:**
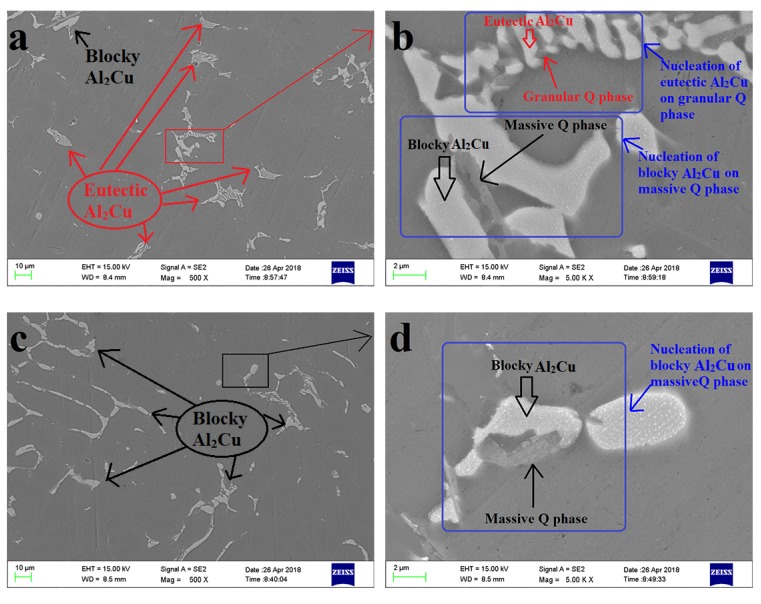
Microstructure of the AlSi9Cu3Mg0.2 and AlSi9Cu3Mg0.6 alloys in the as-cast conditions. (**a**) The morphology and size of Al_2_Cu in the AlSi9Cu3Mg0.2, (**b**) amplification of (**a**) red rectangular area, (**c**) the morphology and size of Al_2_Cu in the AlSi9Cu3Mg0.6, and (**d**) amplification of (**a**) black rectangular area.

The increase of Mg did not effectively change the type of soluble phases but obviously changed the area ratio of the soluble phases, which could be seen from the measurement of the exothermic peak area of the soluble phases in alloys M1, M2, and M3 (Table 5). Compared with alloy M1, when the content of Cu increased from 1.2 wt% to 3 wt% in alloys M2, the increment of the area of endothermic peak A (eutectic Al_2_Cu) was decreased from 19.13 J/g to 15.37 J/g, and the increment of the area of endothermic peak B (blocky Al_2_Cu) was increased from 3.39 J/g to 6.28 J/g. For alloy M3, the increment of the area of endothermic peak A (eutectic Al_2_Cu) was decreased from 19.13 J/g to 14.87 J/g, and the increment of the area of endothermic peak B (blocky Al_2_Cu) was increased from 3.39 J/g to 7.44 J/g. The microstructure of the Al–9Si–3Cu–0.2Mg alloy (Figure 2a) and Al–9Si–3Cu–0.4Mg alloy (Figure 2c) also demonstrated the difference in area ratio between eutectic Al_2_Cu and blocky Al_2_Cu. In the Al–9Si–3Cu–0.2Mg alloy (Figure 2a), most of the Al_2_Cu was the eutectic type, while in the Al–9Si–3Cu–0.4Mg alloy (Figure 2c), blocky Al2Cu became the main type. The type of Al_2_Cu was affected by morphology and size of the Q phase, which provided a nucleation site for Al_2_Cu [15,28]. In the Al–9Si–3Cu–0.2Mg alloy (Figure 2b), a large amount of eutectic Al_2_Cu nucleated on small-size granular Q-Al_5_Cu_2_Mg_8_Si, and a small amount of blocky A_2_Cu nucleated on large-size massive Q-Al_5_Cu_2_Mg_8_Si, while in the Al–9Si–3Cu–0.4Mg alloy (Figure 2d), only blocky Al_2_Cu nucleated on massive Q-Al_5_Cu_2_Mg_8_Si, and no grained Q-Al_5_Cu_2_Mg_8_Si was found. At the Mg-rich phase, the morphology and size of Q-Al_5_Cu_2_Mg_8_Si were dependent on the Mg content. In all, a 0.2 wt% addition of Mg limited the growth of Q-Al_5_Cu_2_Mg_8_Si and showed small-size granular morphology, which provided a nucleation site for the fine eutectic Al_2_Cu. When Mg > 0.2 wt%, enough Mg atoms facilitated the growth of the Q phase and appeared with a large-size massive morphology, which provided a nucleation site for blocky Al_2_Cu. 

#### 3.1.2. Effect of Cu and Mg Contents on the Mechanical Properties of Al–Si–Cu–Mg Alloy

**Figure 3:**
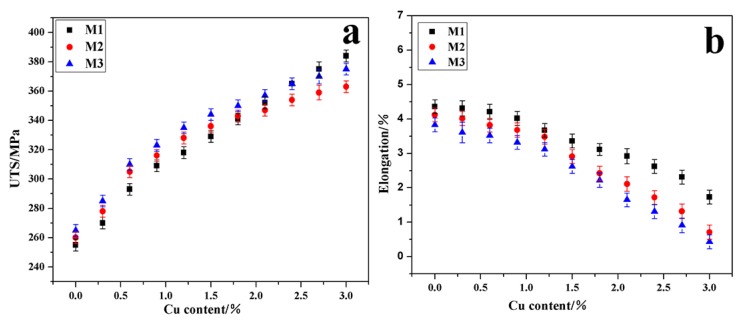
Evolution of mechanical properties of alloys M1, M2, and M3 in the heat treatment. (**a**) Ultimate tensile strength (UTS), (**b**) elongation to fracture (E%).

The evolution of the ultimate tensile strength (UTS) and elongation to fracture (E%) of alloys M1, M2, and M3 after heat treatment are shown in Figure 3a,b. When Cu ≤ 1.2 wt%, the strengthening responses per added 0.1 wt% Cu of alloys M1, M2, and M3 were similar at 5.2 MPa, 5.7 MPa, and 5.8 MPa, respectively. Meanwhile, the responses of elongation reduction per added 0.1 wt% Cu were also similar, with 0.054%, 0.057%, and 0.059%, respectively. When Cu > 1.2 wt%, the strengthening response per added 0.1 wt% Cu of the three alloy systems was weakened (especially the alloys M2 and M3), with 4.4 MPa, 2.3 MPa, and 2.6 MPa, respectively. The response of elongation reduction per added 0.1 wt% Cu of the three alloy systems was enhanced (especially the alloys M2 and M3), with 0.104%, 0.154%, and 0.149%, respectively.

The occurrence of such differences was directly related to the area ratio of the blocky Al_2_Cu phase in the three alloys. Sjölander [29] studied the dissolution of Al_2_Cu in Al–Si–Cu–Mg alloys and found that the complete dissolution time of the blocky Al_2_Cu was more than ten times that of the eutectic Al_2_Cu. The existence of the blocky Al_2_Cu phase seriously affected the comprehensive mechanical properties of Al–Si–Cu–Mg alloys. On the one hand, Cu atoms were occupied by blocky Al_2_Cu and could not be dissolved into the Al matrix, which weakened the ageing strengthening response. On the other hand, the blocky structure easily became the crack source during loading, which led to a decrease in ductility [15,16]. According to Table 5, when Cu ≤ 1.2 wt%, the area of exothermal peak B corresponding to the blocky Al_2_Cu in the three alloy systems was very small. A large number of Cu atoms was mainly concentrated in eutectic Al_2_Cu, so the blocky Al_2_Cu had little effect on the poisoning of the mechanical properties. However, when Cu > 1.2 wt%, the area of exothermic peak B was effectively increased in the three alloys, especially in alloys M2 and M3. Therefore, the negative effects of blocky Al_2_Cu on the mechanical properties of the three alloys were highlighted. To avoid the formation of large amounts of blocky Al_2_Cu and to ensure excellent strength–ductility coordination, Al–9Si–1.2Cu–0.4Mg (UTS of ~328 and E% of ~3.48%) was selected as the optimized base alloy with the best matches of Cu and Mg.

### 3.2. Modification of Insoluble Phases by Zn in the Al–9Si–1.2Cu–0.4Mg-Based Alloy

#### 3.2.1. Effect of Zn Content on Insoluble Phases of Al–Si–Cu–Mg-Based Alloy in As-Cast Conditions

**Figure 4:**
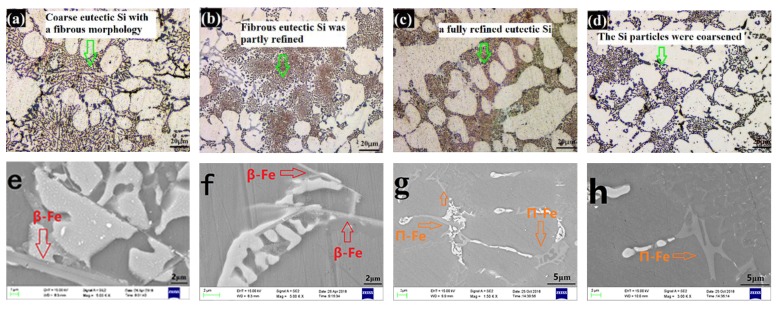
The morphology of the eutectic Si by OM and the size and texture of Fe-rich phases by SEM of Al9Si1.2Cu0.4Mg (0 wt%–0.9 wt% Zn) in as-cast conditions: (**a**,**e**) free-Zn, (**b**,**f**) 0.3 wt% Zn, (**c**,**g**) 0.6 wt% Zn, (**d**,**h**) 0.9 wt% Zn.

The morphology of the eutectic Si obtained by OM of Al–9Si–1.2Cu–0.4Mg (0 wt%–0.9 wt% Zn) in as-cast conditions is shown in Figure 4a–d. As seen from Figure 4a, the base alloy exhibited coarse eutectic Si with a fibrous morphology. The size of the fibrous eutectic Si was partly refined with the addition of 0.3 wt% Zn, as seen in Figure 4b. The addition of 0.6 wt% Zn resulted in a fully refined eutectic Si, and consequently, Si particles showed a finer fibrous content, as shown in Figure 4c. When 0.9 wt% Zn was added, the Si particles became coarser (Figure 4d). 

The size and texture of Fe-rich phases of Al–9Si–1.2Cu–0.4Mg (0 wt%–0.9 wt% Zn) in as-cast conditions as determined by SEM are displayed in Figure 4e–h. For the base alloy (Figure 4e) and 0.3 wt% Zn modified alloys (Figure 4f), the acicular β-Al_5_FeSi was the main form of Fe-rich phases (red arrows), but the size of β-Al_5_FeSi in the base alloy was larger than that in the 0.3 wt% Zn modified alloy. Compared with the base alloy and the 0.3 wt% Zn modified alloys, the Chinese π-Fe was the main form of Fe-rich phases (yellow arrows) in the 0.6 wt% Zn modified (Figure 4g) and 0.9 wt% Zn modified (Figure 4h) alloys. However, the size of π-Fe in the 0.9 wt% Zn modified alloy was larger than that in the 0.6 wt% Zn modified alloy.

The modification of insoluble phases (eutectic Si and β-Al5FeSi) by Zn could be attributed to the reaction of eutectic Zn–Si (419.58 °C) [30]. For the insoluble phases, both the eutectic Si and β-Al_5_FeSi phases had growth tendencies on the {111}_Si_ planes [17]. However, Zn atoms tended to segregate on the {111}_Si_ planes during solidification [31]. The enrichment of Zn atoms on the {111}_Si_ planes resulted in the continuous reaction of Si atoms in the insoluble phases (eutectic Si and β-Al_5_FeSi) with Zn atoms and forced Si atoms into eutectic droplets. The reaction of eutectic Zn–Si limited the growing trend of eutectic Si and β-Al_5_FeSi on {111}_Si_ planes, which limited the growth of insoluble phases. As opposed to the single type of eutectic Si, Fe-rich phases have multiple textures. When the growth of the β-Al_5_FeSi phase is limited, the redundant Fe atoms prefer to form a π-Fe phase with Mg atoms. When 0.3 wt% Zn was added, the low amount of Zn atoms made it difficult for them to fully react with the Si atoms in the insoluble phases, which resulted in the size of the fibrous eutectic Si being only partly refined; thus, the β-Fe phase could not be modified to the π-Fe phase. When 0.6 wt% Zn was added, the moderate amount of Zn atoms was segregated on the {111}_Si_ planes, resulting in the full reaction of eutectic Zn–Si. Thus, the size of the fibrous eutectic Si was fully refined, and the acicular β-Fe was modified to the Chinese π-Fe phase. When 0.9 wt% Zn was added, the excess Zn atoms became the transport solvent of Si atoms between the insoluble phases. Due to the small spacing between the insoluble phases, especially the eutectic Si particles, multiple insoluble phases were gathered together during solidification. This was also the reason why the size of the eutectic Si and π-Fe phase was increased by the addition of 0.9 wt% Zn.

#### 3.2.2. Effect of Zn Content on the Insoluble Phases of Al–Si–Cu–Mg Alloy in the Heat Treatment

**Figure 5:**
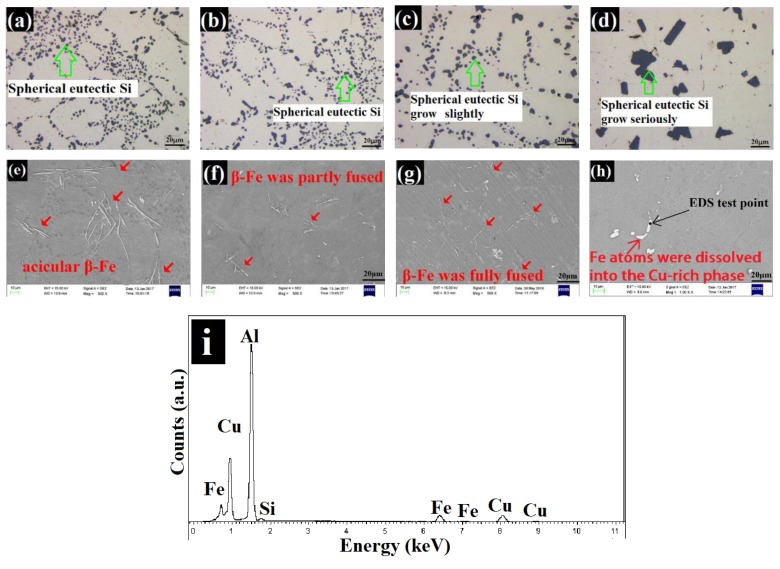
The morphology of the eutectic Si by OM and the size and texture of Fe-rich phases, determined by SEM, of Al9Si1.2Cu0.4Mg (0 wt%–0.9 wt% Zn) in the heat treatment: (**a,e**) free-Zn, (**b,d,f**) 0.3 wt% Zn, (**c,g**) 0.6 wt% Zn, (**d,h**) 0.9 wt% Zn, (**i**) EDS detection of (**h**).

In the heat treatment, although eutectic Si could not be dissolved in the Al matrix, its morphology and size were affected by the thermal environment. The evolution of the eutectic Si during heat treatment was as follows: (1) fusing, (2) becoming spherical, and (3) coarsening [15]. The morphology of the eutectic Si of Al–9Si–1.2Cu–0.4Mg (0 wt%–0.9 wt% Zn) in the heat treatment, as determined by OM, is shown in Figure 5a–d. For the base alloy and 0.3 wt% Zn modified alloys (Figure 5a,b), the size of the spherical eutectic Si was similar. For 0.6 wt% Zn modified alloys (Figure 5c), although the eutectic Si remained spherical, its size was slightly larger than that of the base alloy and 0.3 wt% Zn modified alloy. For the 0.9 wt% Zn modified alloy (Figure 5d), excessive Zn modifier caused the eutectic Si to substantially grow and exist in blocky morphology. 

As opposed to the process of fusing, becoming spherical, and coarsening of eutectic Si, the β-Fe with high thermal stability only partly fused during solution treatment. The fusing degree of the β-Fe phase was affected by its size and solution temperature [16]. The size and texture of the Fe–rich phases of Al–9Si–1.2Cu–0.4Mg (0 wt%–0.9 wt% Zn) in the heat treatment, determined by SEM, are shown in Figure 5e–h. Compared with the as-cast condition, the heat treatment did not change the morphology or size of β-Fe in the base alloy (Figure 5e). However, for the 0.3 wt% Zn modified alloy (Figure 5f), the acicular β-Fe was partly fused. In the 0.6 wt% Zn modified alloy, π-Fe disappeared after the heat treatment, and fully fused β-Fe was observed (Figure 5g). In the 0.9 wt% Zn modified alloy (Figure 5h,i), π-Fe disappeared after the heat treatment, and Fe atoms were dissolved into the Cu-rich phase. Sjölander [8] noted that the π-Fe with poor thermal stability decomposed into tiny β-Fe and Mg atoms in the early stage of solution treatment. This is also the reason why π-Fe disappeared in 0.6 wt%–0.9 wt% Zn modified alloys. Compared with the large-size β-Fe in the base alloy, the original fine β-Fe in 0.3 wt% Zn modified alloy or the very fine β-Fe transformed by the π-Fe phase in 0.6 wt%–0.9 wt% Zn modified alloy were more likely to be fused or dissolved during the solution treatment.

#### 3.2.3. Effect of Zn Content on the Mechanical Properties of Al–Si–Cu–Mg Alloy during Heat Treatment

**Figure 6:**
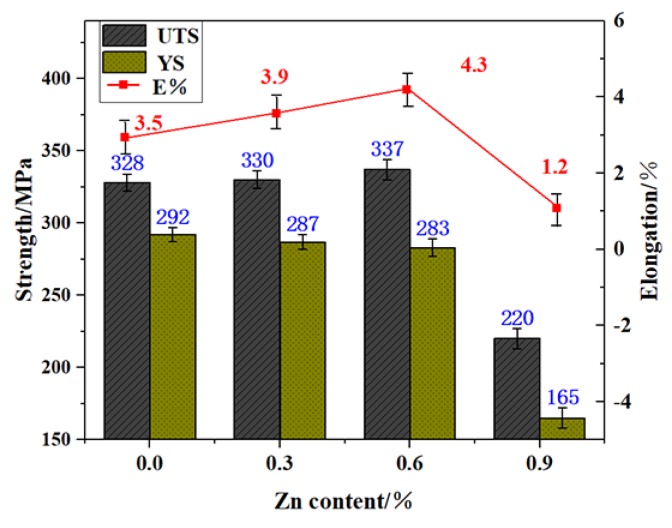
Effect of Zn content on the mechanical properties of Al–9Si–1.2Cu–0.4Mg-based alloys during the heat treatment.

The mechanical properties of Al–Si–Cu–Mg alloys depend largely on the second phases, especially on the eutectic Si and Fe-rich phases, which tend to cause stress concentration and eventually become the origin of cracks when stressed. Figure 6 shows the effect of Zn content on the UTS, yield strength (YS), and E% of Al–9Si–1.2Cu–0.4Mg-based alloys during heat treatment. The results indicated that when the content of Zn increased from 0 wt% to 0.6 wt%, the UTS and E% were enhanced from 328 MPa and 3.48% to 337 MPa and 4.31%, respectively, but the YS decreased slightly from 292 MPa to 283 MPa. The improvement of the UTS and E% could be attributed to two aspects. On the one hand, a large number of fused β-Fe existed in Zn modified alloys. The fused β-Fe reduced the brittle fracture caused by stress concentration and improved the E%. On the other hand, Zn addition resulted in a higher density of precipitates and improved the distribution of heterogeneous solute-rich features in artificial ageing, as indicated by Guo et al. [32,33,34,35]. The homogeneous and high-density precipitates effectively blocked the dislocation movement during the loading and improved the UTS. In terms of the YS, the Zn addition caused the β-Fe to be fused during solution treatment, which destroyed the tightly locked relationship between the hard phases and the soft Al matrix. Thus, the YS decreased. Based on the effect of the Zn modifier on the insoluble phase and mechanical properties of the Al–Si alloys, Al–9Si–1.2Cu–0.4Mg–0.6Zn (UTS ~337 MPa, YS ~283 MPa, and E% ~4.31%) was selected as the optimized modified alloy for the following ageing experiments.

### 3.3. Optimization of Artificial Ageing Processes in Base Alloy and 0.6 wt% Zn Modified Alloy

#### 3.3.1. Effect of Zn on the Ageing Curve of Al–Si–Cu–Mg Alloy

**Figure 7:**
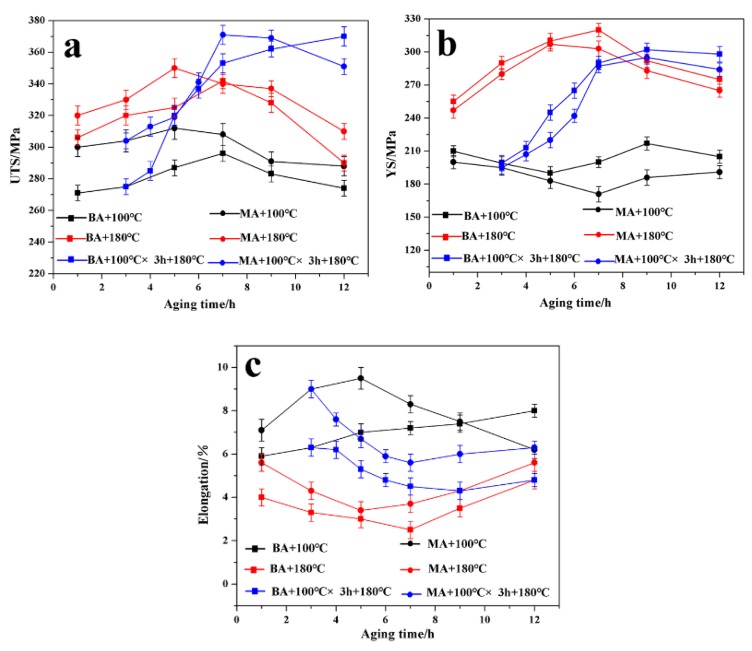
Evolution of mechanical properties of Al–9Si–1.2Cu–0.4Mg–0.6Zn modified alloy and Al–9Si–1.2Cu–0.4Mg-based alloy under three types of artificial ageing processes: (**a**) UTS, (**b**)YS, (**c**)E% (BA—Base alloy, MA—0.6 wt% Zn modified alloy).

The evolution of the mechanical properties of the Al–9Si–1.2Cu–0.4Mg–0.6Zn modified alloy under three types of artificial ageing processes is shown in Figure 7. For comparison, the Al–9Si–1.2Cu–0.4Mg-based alloy is also presented in Figure 7. Comparing the mechanical property curves of the base alloy and the 0.6 wt% Zn modified alloy under three types of artificial ageing processes, it can be seen that the UTS and E% of the 0.6 wt% Zn modified alloy were generally higher than those of the base alloy under the same ageing parameters, but the YS was generally lower than that of the base alloy. This is consistent with the results shown in Figure 6, which were mainly due to the existence of fused β-Al_5_FeSi and a high-density precipitate phase in the 0.6 wt% Zn-modified alloy [32,33,34,35]. Comparing the peak ageing of the base alloy and the 0.6 wt% Zn modified alloy, it can be seen that the time needed to obtain the peak strength of the Zn modified alloy was significantly shorter than that of the base alloy under the same ageing process. This result shows good agreement with Guo et al. [33] for the precipitation behaviors of Al–Mg–Si–Cu alloys with different Zn contents. They established the kinetics equations of GP zone dissolution and precipitation, and the calculation results revealed that the precipitation rates of the natural aged or pre-aged alloys can be improved by Zn.

#### 3.3.2. Effect of Artificial Ageing Processes on Mechanical Properties of Base Alloy and 0.6 wt% Zn Modified Alloy

**Figure 8:**
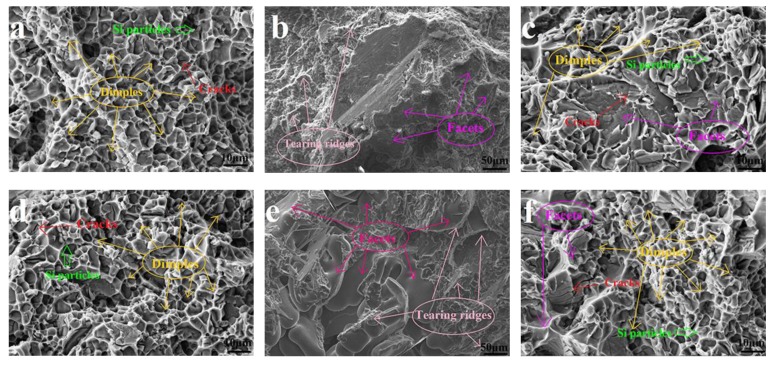
Fracture surface of the base alloy and the 0.6 wt% Zn modified alloy in three types of ageing processes at peak ageing: (**a**) base alloy—100 °C × 7 h, (**b**) base alloy—180 °C × 7 h, (**c**) base alloy—100 °C × 3 h + 180 °C × 9 h, (**d**) 0.6 wt% Zn modified alloy—100 °C × 5 h, (**e**) 0.6 wt% Zn modified alloy—180 °C × 5 h, (**f**) 0.6 wt% Zn modified alloy—100 °C × 3 h + 180 °C × 4 h.

During single-stage low-temperature ageing (100 °C), the strengths of the base alloy and the 0.6 wt% Zn modified alloy were not effectively improved with increasing ageing time, but the E% remained at a high level. Figure 8a,d shows the fracture surface of the base alloy and the 0.6 wt% Zn modified alloy in peak ageing (E% ~7.2% and 9.5%, respectively). Most of the fracture surfaces of the two alloys were occupied by dimples, and there were few facets. It was also found that most of the granular eutectic Si particles (green arrows) were pulled out in dimples, and cracks formed in the Al matrix. The Cu and Mg atoms in the supersaturated solid solution diffused to the grain boundary (formation of the GP zone), demonstrating a process of stress release, which required a lower activation energy [25]. The lower temperature was enough to make the solute atoms Cu and Mg overcome the energy barrier to form the GP zone. The formation of fine and uniform GP zones could effectively promote the E% of the base and modified alloys. Compared with the formation of the GP zone, the formation of metastable precipitates required a higher activation energy to cross the energy barrier [26]. The strength of the alloy could not be effectively improved because the formation of metastable precipitates was limited by the low ageing temperature.

During single-stage high-temperature ageing (180 °C), the strengths of the base and the 0.6 wt% Zn modified alloys were effectively improved with increasing ageing time, but the E% was low. Figure 8b,e shows the fracture surfaces of the base alloy and the Zn modified alloy during peak ageing (E% 2.5% and 3.4%, respectively). Most of the fracture surfaces of the two alloys were occupied by facets and tearing ridges; there were few dimples. Compared with low-temperature ageing, high-temperature ageing could provide sufficient energy to promote the formation of metastable precipitates and stable precipitates, which led to an increase in strength and a decrease in E%.

Compared with the high-temperature ageing, the two-stage ageing (100 °C × 3 h + 180 °C) process not only improved the strength of the base alloy and the 0.6 wt% Zn modified alloy to a certain extent but also maintained a high level of E%. Figure 8c,f shows the fracture surfaces of the base alloy and the 0.6 wt% Zn modified alloy during peak ageing (E% of ~4.8% and 5.6%, respectively). Compared with the fracture surface of the base and modified alloys during peak ageing at high-temperature ageing, the area ratio of facets decreased, and the area ratio of dimples increased noticeably at double-stage ageing. The improvements of the UTS and E% were attributed to the excellent breeding environment of the GP zone due to the low ageing temperature at the early stage of ageing. Compared with the initial high ageing temperature, the initial low ageing temperature was conducive to the formation of a more uniform and higher-density GP zone. When the ageing temperature increased to 180 °C, the metastable phases began to form in the homogeneous and high-density GP zone. The metastable phases with uniform distribution and high density could not only ensure high strength but could also improve the elongation of the base alloy and the 0.6 wt% Zn modified alloy. Therefore, double ageing of 100 °C × 3 h + 180 °C × 7 h (for Al–9Si–1.2Cu–0.4Mg–0.6Zn alloy with a UTS of ~371 MPa, YS of ~291 MPa, and E% of ~5.6% achieved) was selected as the optimized artificial ageing process.

## 4. Conclusions

Based on the present results, the following conclusions may be drawn:

(1) For the soluble phases, the increase of Cu effectively changed the type of soluble phases from Mg-rich phases to Cu-rich phases, while the increase of Mg did not effectively change the type of soluble phases, but obviously changed the area ratio of soluble phases. Especially when Cu > 1.2 wt% and Mg > 0.2 wt%, the formation of large amounts of blocky Al_2_Cu caused weakening of the strengthening response according to the 0.1 wt% Cu added; 

(2) For the insoluble phases in the as-cast condition, the addition of 0.6 wt% Zn modified the eutectic Si from a coarse fibrous into a fine fibrous state, while the Fe-rich phases were modified from acicular β-Fe to Chinese π-Fe by the addition of 0.6 wt% Zn. Adding more Zn (i.e., 0.9 wt%) made the eutectic Si coarser, and the Fe-rich phases still retained the Chinese π-Fe phase;

(3) For the insoluble phases of the heat treatment, the addition of 0.6 wt% Zn resulted in slight increases in the size of spherical eutectic Si, while the fine β-Fe phase transformed by the π-Fe phase was fully fused to a smaller size. The addition of more Zn (i.e., 0.9 wt%) resulted in substantial growth of eutectic Si with a blocky structure, and Fe atoms were dissolved into the Cu-rich phase; 

(4) Compared with the base alloy, the 0.6 wt% Zn modified alloy not only reduced the time of peak ageing but also increased the value of peak ageing. A better combination of strength and ductility was achieved for both the base alloy and the 0.6 wt% Zn modified alloy by double-stage ageing; 

(5) Based on the Al–Si alloy, the 1.2 wt% Cu and 0.4 wt% Mg, 0.6 wt% Zn modifier, and double-stage ageing at 100 °C × 3 h + 180 °C × 7 h were selected as the optimized parameters with not only the best matches of Cu and Mg contents but also the most suitable artificial ageing processes, with improved mechanical properties of UTS of ~371 MPa, YS of ~291 MPa, and E% of ~5.6% obtained.

## Figures and Tables

**Table 1:** Change in Mg element in alloy composition in wt%.

Alloy	Si	Cu	Mg	Fe	Ti	Sr	Al
M1	8.8	Change	0.21	0.16	0.16	0.0054	Bal.
M2	9.1	Change	0.38	0.15	0.17	0.0061	Bal.
M3	8.9	Change	0.57	0.18	0.15	0.0046	Bal.

**Table 2:** Change in Cu in alloy composition in wt%.

Alloy	Change in Cu Content (%)										
M1	0.03	0.31	0.61	0.94	1.21	1.51	1.81	2.11	2.41	2.69	3.01
M2	0.01	0.29	0.61	0.93	1.17	1.51	1.83	2.07	2.41	2.71	3.03
M3	0.04	0.28	0.63	0.94	1.19	1.52	1.81	2.09	2.39	2.72	2.98

**Table 3:** Change of Zn element in alloy composition in wt%.

Alloy	Si	Cu	Mg	Zn	Fe	Ti	Sr	Al
Al–9Si–1.2Cu–0.4Mg (0.005 Sr)	8.7	0.93	0.41	-	0.14	0.14	0.0050	Bal.
Al–9Si–1.2Cu–0.4Mg (0.3 Zn + 0.005 Sr)	8.8	0.95	0.39	0.29	0.15	0.16	0.0052	Bal.
Al–9Si–1.2Cu–0.4Mg (0.6 Zn + 0.005 Sr)	9.1	0.94	0.38	0.61	0.15	0.15	0.0056	Bal.
Al–9Si–1.2Cu–0.4Mg (0.9 Zn + 0.005 Sr)	8.9	0.92	0.42	0.88	0.16	0.15	0.0054	Bal.

**Table 4:** Reaction of endothermic peaks deduced by Mondolfo [27].

Peak	Reaction	Temperature
Endothermic peak A	Eutectic Al_2_Cu→liquid	507 °C
Endothermic peak B	Blocky Al_2_Cu→liquid	520 °C
Endothermic peak C	Al_5_Cu_2_Mg_8_Si_6_→liquid	534 °C
Endothermic peak D	Mg_2_Si→liquid	554 °C
Endothermic peak E	Eutectic Si→liquid	569 °C

**Table 5:** The influence of the evolution of Cu and Mg composition (wt%) on **t**he area of each endothermic peak (J/g).

Area of Peak		Peak A			Peak B			Peak C			Peak D		
	Mg	0.2 Mg	0.4 Mg	0.6 Mg	0.2 Mg	0.4 Mg	0.6 Mg	0.2 Mg	0.4 Mg	0.6 Mg	0.2 Mg	0.4 Mg	0.6 Mg
Cu													
0 Cu		-	-	-	-	-	-	-	-	-	1.43	1.64	1.75
0.6 Cu		0.76	0.38	0.31	-	-	-	0.53	0.41	0.51	-	0.78	0.96
1.2 Cu		2.32	2.21	2.11	0.73	0.89	0.87	0.96	1.08	1.33	-	-	-
1.8 Cu		6.14	4.53	4.02	1.91	2.95	3.02	-	-	-	-	-	-
2.4 Cu		16.71	9.54	8.99	2.97	5.61	7.72	-	-	-	--		-
3 Cu		21.45	17.54	16.98	4.12	7.17	8.31	-	-	-	-	-	-
